# Supplementary material for: Knockout of Purinergic P2Y6 Receptor Fails to Improve Liver Injury and Inflammation in Non-Alcoholic Steatohepatitis
Source: Int J Mol Sci. 2023 Feb 14;24(4):3800. doi: 10.3390/ijms24043800 (PMC9963899; doi:10.3390/ijms24043800)
Supplement: Supplementary file 1 [file ijms-24-03800-s001.zip › ijms-2154491-supplementary.pdf]

supplementary Table S1. Primers

gene	forward (5′–3′)	reverse (5′–3′)
P2Y6R	GTGAGGATTTCAAGCGACTGC	TCCCCTCTGGCGTAGTTATAGA
CCL2	TTAAAAACCTGGATCGGAACCAA	GCATTAGCTTCAGATTTACGGGT
IL-6	AAGGGCCAGGGATCTGTAAG	TCTCTTGTTGCTCCCCAAAG
TGFβ1	CTCCCGTGGCTTCTAGTGC	GCCTTAGTTTGGACAGGATCTG
Col1a1	GCTCCTCTTAGGGGCCACT	CCACGTCTCACCATTGGGG
18s rRNA	ATTAATCAAGAACGAAAGTCGCAGGT	TTTAAGTTTCAGCTTTGCAACCATACT
